# Inkjet-Printed Functionalization of CMUT-Based CO_2_ Sensors

**DOI:** 10.3390/s22062288

**Published:** 2022-03-16

**Authors:** Dovydas Barauskas, Donatas Pelenis, Mindaugas Dzikaras, Marius Mikolajunas, Gailius Vanagas, Darius Virzonis

**Affiliations:** Panevezys Faculty of Technology and Business, Kaunas University of Technology, 44249 Kaunas, Lithuania; dovydas.barauskas@ktu.lt (D.B.); donatas.pelenis@ktu.lt (D.P.); mindaugas.dzikaras@ktu.lt (M.D.); marius.mikolajunas@ktu.lt (M.M.); gailius.vanagas@ktu.lt (G.V.)

**Keywords:** carbon dioxide sensing, capacitive micromachined ultrasound transducers, polyethyleneimine, silicon micromachining, inkjet printing, surface functionalization

## Abstract

The trade-off between the functionalization shift of the informative parameters and sensitivity of capacitive micromachined ultrasound transducers (CMUT)-based CO_2_ sensors is addressed, and the CMUT surface modification process by thin inkjet-printed polyethyleneimine (PEI) films is optimized. It was shown that by the proper preparation of the active CMUT surface and properly diluted PEI solution, it is possible to minimize the functionalization shift of the resonance frequency and the quality of the resonance and preserve the sensitivity potential. So, after optimization, we demonstrated 23.2 kHz frequency shift readings of the sensor with 16 MHz nominal frequency while in the gas chamber and switching between pure N_2_ and CO_2_. After testing the sensors with different PEI film thickness, it was confirmed that a 200 nm average thickness of a PEI film is an optimum, because this is the practical limit of CO_2_ absorption depth at given conditions. Additionally, we note that modification of the hydrophilic/hydrophobic properties of the CMUT surface allows changing the nanoscale surface roughness of the printed PEI film and controlling the area resolution of the inkjet functionalization by reducing the diameter of a single dot down to 150 μm by a commercially available printer cartridge.

## 1. Introduction

Control over the anthropogenic emissions of greenhouse effect gases, such as carbon and sulfur dioxides, methane, and some others [1], is a crucially important measure to slow down global warming [2]. This calls for the development of affordable gas sensors with improved sensitivity, cross-selectivity, and reliability. There are many different gas-sensing technologies in the market that are commercially available; however, recent developments in MEMS (microelectromechanical systems) technology led to a widespread wave of innovation and research in micro- and nanoscale electromechanical gas-sensing devices [3,4]. When comparing these emerging gas-sensing technologies in the terms of selectivity, sensitivity, associated cost, footprint, and response time, the CMUT (capacitive micromachined ultrasound transducers) approach to gas sensing is apparently attractive due to the low cost of mass production and the possibilities of making highly selective and sensitive devices with fast response times that are small, portable, and very attractive for integration into mobile applications [5]. Earlier works published by Khuri-Yakub et al. (Stanford University) demonstrated the unprecedented sensitivity of CMUT-based CO_2_/humidity sensors thanks to the sophisticated functionalization of the device surface with a mesoporous silica film and (3-aminopropyl) triethoxysilane (APTES) [6] as well as with a guanidine-based polymer [7]. There was also an interesting development by Thomsen et al. (Technical University of Denmark), demonstrating the CMUT-based combined colorimetric and gravimetric detection of gaseous organic compounds [8]. Humidity sensors developed by Yeow et al. (University of Waterloo) employ nanocrystalline cellulose and graphene oxide as moisture-absorbing functional materials for CMUTs [9,10]. Our group has recently published proof of concept research on gas sensors for CO_2_, SO_2_, and their mixtures based on polyethyleneimine (PEI) and methylated polyethyleneimine (mPEI) functionalized capacitive micromachined ultrasound transducers (CMUTs) [11,12,13]. In our previous experiments and the works of some other groups, the polymer functional films were applied over the CMUT surface with different techniques, particularly spin coating, dip coating, spray coating, drop coating, and some others. Although these are suitable techniques for the essential sensor functionality, there are significant drawbacks, such as the functional material waste, comparatively low technological feasibility, and repeatability of the functionalization results. The modification of CMUT microstructures by the application of an imprecisely controlled amount of functional material is subject to various physical effects, which lead to uncontrollable thickness variations of the functional film. In addition, the abovementioned processes are bulky and require careful maintenance from skilled personnel. For the reliable and repeatable operation of a functionalized sensor, the functional film thickness is to be optimized for a minimum load and damping of the CMUT structure and for the maximum sensitivity. Some alternative coating technologies are less suitable for the selected functional materials or, as in the case of drop coating, have even lower technological reliability potential and hence lower potential for control over the resulting technical parameters of the sensor. One of the most promising functional film deposition techniques is inkjet printing, which was recently reported to be successfully applied on CMUT moisture sensors with graphene oxide as a functional material [9,10,14]. In addition, the inkjet printing of polymer materials is already well known and accepted in the fabrication of microelectromechanical devices [15]. In addition to the improved control over the functional film morphology and thickness, inkjet technique also provides the ability to selectively deposit different functional films to different areas of the sensor. Differently functionalized sensor areas are essential for multichannel gas sensing and for improved cross-selectivity [6,11]. In addition, due to the need for sensor cost reduction, the silicon area dedicated to specific gas molecules must be kept to a minimum. Here, the control over the inkjet-printed pattern dimensions becomes important. In general, the size and morphology of the printed functional film pattern is dependent not only on the function of the ejected droplet size but also on the functional material and the device surface properties. As reported in recent research, the resolution of the inkjet-printed graphene oxide film pattern deposited on a device can be 300 μm [9,14], which can be considered too rough for the densely packed CMUT membrane elements of multichannel gas sensors. Therefore, in our research, we explore the interdependence of the device surface preparation technique and the resulting pattern of the printed functional film. Furthermore, we have tested and optimized the inkjet-printed PEI films for the response of fabricated sensors to CO_2_ gas.

## 2. Materials and Methods

### 2.1. Functional Material and Thickness Control

We used polyethylenenimine (PEI) as a functional material. PEI (average Mn 1200, average Mw 1300, 50% *w*/*v* in H_2_O), methanol, isopropyl alcohol (IPA), and acetone were purchased from Sigma Aldrich (US). Hexamethyldisilizane (HMDS), used for surface preparation, was purchased from Microchemicals GmbH (Germany). For different experiments, we used diluted PEI solutions with methanol of 0.3% *w*/*v* and 1% *w*/*v*. Dilution of PEI was our primary tool to control the viscosity of the solution to meet the specifications of the inkjet cartridge and to variate the thickness of the resulting functional film.

Optical microscopy (Nikon Eclipse L200N) in coordination with atomic force microscopy (AFM Nanosurf EasyScan 2) working in tapping mode was used to measure the deposited functional film thickness through light interference and film color. See the Experimental section for detailed description of the average film thickness measurement.

### 2.2. Inkjet Printing Technique

**Figure 1:**
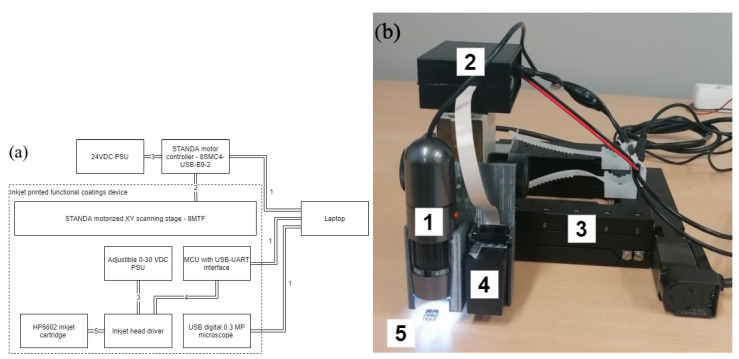
Inkjet printing system; (**a**) block diagram: 1—serial communication; 2—stepper motor cable; 3—power cable; 4—control cable; 5—cartridge cable; (**b**) a photograph of the system: 1—camera, 2—printing controller and driver, 3—two-axis positioning system, 4—cartridge, 5—CMUT device.

In our experiments, we used inkjet cartridges HP6602A mounted on a lateral two-axis positioning system with the following technical properties: travel range—102 mm × 102 mm; displacement step resolution—2.5 μm with the possibility of increasing the displacement resolution to 0.31 μm if a closed loop system with positioning feedback through encoders was used. The inkjet cartridge was operated by the Atmega328 microcontroller with dedicated firmware. As shown in Figure 1a, microcontroller outputs were interfaced to the cartridge by 500 mA current drivers and an external 18 VDC voltage source. The inkjet printing system was controlled from a PC by serial communication. Another serial interface was used for the optical feedback system—a 0.3 MP USB camera—that was mounted on the positioner near the cartridge. An overall view of the printing system with blocks marked by numbers is shown in Figure 1. The dispensing of material from the cartridge was regulated programmatically from 1 to 1000 droplets for a single cartridge position. After the required volume of material was disposed, the inkjet printer cartridge was moved to the next position and the deposition could be repeated.

Prior to the inkjet printing of PEI, sensor surfaces were prepared by cleaning in deionized water, acetone, and IPA baths for 5 min with light stirring. Afterwards, some surfaces were subject to one or more of the procedures listed below.

1. Dehydration bake—surface activation by dehydration bake was completed on a KarlSuus (Germany, Gmbh) hot plate at 220 °C kept for 60 min.
2. Treatment with HMDS—surface activation by HMDS was completed in a two-step process: firstly, the samples were dehydration baked at 220 °C for 60 min, and then, a thin layer of HMDS primer was spin coated at 2000 rpm for 60 s.
3. O_2_ plasma cleaning and activation—samples were loaded into an Advanced Vacuum reactive ion etching system Vision 320 RIE, where they were subject to oxygen plasma with power of 80 W with O_2_ flow of 40 sccm in 30 mTorr pressure for 60 s.

### 2.3. CMUT Design, Fabrication, and Testing

**Figure 2:**
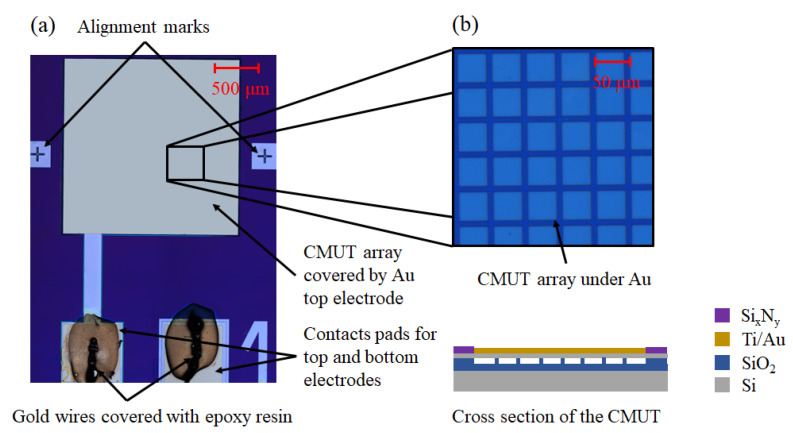
(**a**) Micrograph of the CMUT device showing the array, contact pads for top and bottom electrodes, and the alignment marks used in photolithography steps and for inkjet printing alignment; (**b**) micrograph of the CMUT array under the top electrode and its cross section representation.

CMUTs were fabricated with wafer bonding technology, as described in our earlier publications [11,12], which is promptly outlined here for the reader’s convenience. Highly doped silicon wafers were oxidized with 300 nm of SiO_2_. The vacuum cavities were formed by a photolithography step by etching the oxide layer. Then, silicon on insulator (SOI) wafers with a 2.5 μm device layer thickness and a device layer thickness variation of up to ±0.5 μm over the whole wafer area were prepared to be bonded to the wafers with etched cavities. The prebonding was completed in an SB6 substrate bonder from Suss MicroTec in vacuum at 200 °C and 5 kN force for 30 min. Later, prebonded wafers were transferred into a furnace for annealing for 2 h at 1100 °C. After annealing, the handle side of the SOI wafers were chemically–mechanically polished (CMP Logitech PM5), leaving a thin layer of 100 μm. The remaining part was wet etched in a KOH bath 40 wt % at 80 °C. After removal of the handle wafer, the buried oxide (BOX) layer was wet etched in a buffered oxide etch bath. Another photolithography step and an Oxford cryogenic etching process were completed to separate devices by etching the device layer. Third lithography was used to form openings for contact pads with a reactive ion etching (RIE) procedure. Another lithography step was used to form the required patterns to make the top electrode and the metallization of contact pads with 25 nm of Ti combined with 175 nm of Au thin films, using the lift-off procedure. Lastly, a protective layer of 200 nm PECVD silicon nitride was deposited, and then, the last photolithography step was used to open and reveal the contact areas for wire bonding by etching the deposited Si_x_N_y_ layer in the contact pad areas. A micrograph of a fabricated CMUT device with explanatory cross-sectional drawing is shown in Figure 2.

The surface picture of wafer-bonded CMUT does not display the pattern of individual cells, since they are under the cover of non-transparent Ti/Au film. The underlying structure is explained at the right of the micrograph, showing the view from above to the part of an array of CMUT cells and the drawing of a vertical cross-section, which is shown not to scale for improved visual representation. Square CMUT cells have a side length of 42 μm and a pitch of 50 μm in between them. The thickness of a CMUT monocrystal silicon membrane is 2.5 ± 0.5 μm. The CMUT cell array is coated with 200 nm thick Ti/Au film from the top of the membranes for improved conductivity and adhesion with the functional material. This design was targeted for 16 MHz resonance in the normal conditions (atmospheric pressure, ambient air, 45% humidity, 20 °C) and 0 V bias, though due to the device layer thickness variations, damping of functional coatings, and non-zero bias voltages used in experiments, the actual resonance frequencies presented in the following subsections are different from the nominal resonance frequency featured in the design.

**Figure 3:**
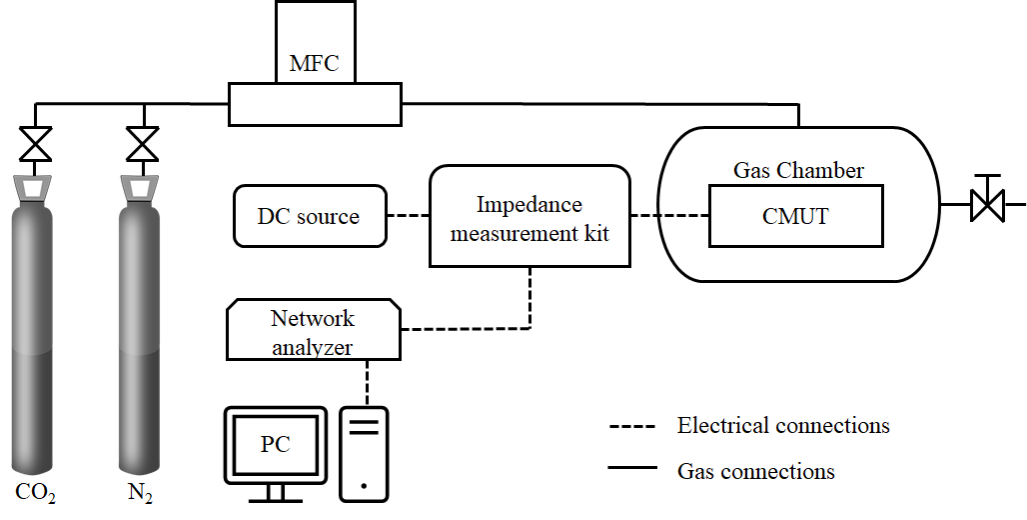
Block diagram of the experimental system setup for sensor testing in CO_2_ and N_2_ gas environments.

Functionalized CMUTs (sensors) were tested in ambient air and in a gas chamber. The setup of the gas chamber and the electromechanical testing system is schematically shown in Figure 3. A CMUT sensor was held inside of the gas chamber exposed to the gas. It was connected through a pair of wires to the testing equipment situated outside of the gas chamber. The network analyzer Agilent 4395A equipped with an impedance measurement kit 43961A in conjunction with a direct current bias voltage source N5752A was used to capture the electromechanical impedance spectra of the sensor.

### 2.4. Optimization of the Printed PEI Thickness

Properties of the functional film deposited over a CMUT structure will affect the performance of the electromechanical part of the sensor by reducing the resonance frequency and (in some cases) degrading the resonance quality. This effect is named here as “functionalization shift”. The functionalization shift can be minimized by reducing the thickness of the functional film. However, as it was revealed from the functionalization experiments in this work, the functional film cannot be too thin, since then, sensor readings (resonance frequency shift due to the absorption of the gas molecules) become compromised with not using the full CO_2_ absorption depth, which is at the range of hundreds of nanometers, according to our recent depth-resolved spectroscopy research [11]. Therefore, the printed PEI film thickness needs to be optimized. As there are two frequency shifts, one of which is to be minimized (the functionalization shift) and another one (sensor reading shift) that is to be kept maximum, a common optimization parameter estimating the ratio between the two frequency shifts can be established:

(1) $$\varsigma =\frac{\Delta f_{SR}}{\Delta f_{func}},$$

where ς is the optimization parameter, $\Delta f_{SR}$—sensor reading shift; $\Delta f_{func}$—functionalization shift. We investigated the optimum of the functional film thickness by the functionalization and testing of series of the sensors. There were five groups of sensors with functional films of six different thickness levels. Thickness differences were achieved by printing an increasing number of functional film layers and by using 1% *w*/*v* and 0.3% *w*/*v* PEI solutions. Each sensor underwent prefunctionalization testing by measurement of the natural resonance frequency in the ambient air, and then, it was functionalized by printing from one to six layers of the functional film. After functionalization, the sensors underwent resonance frequency measurement in the ambient air, and the functionalization shift was calculated. Then, sensors were taken to the gas chamber, and the functionalization shift was measured again in pure nitrogen. After this measurement, the CO_2_ sensing shift (sensor reading, SR shift) was recorded by switching the chamber from pure nitrogen to pure carbon dioxide to have the full-scale sensor readings.

## 3. Results

### 3.1. Thickness and Morphology of the Inkjet-Printed PEI Coatings

**Figure 4:**
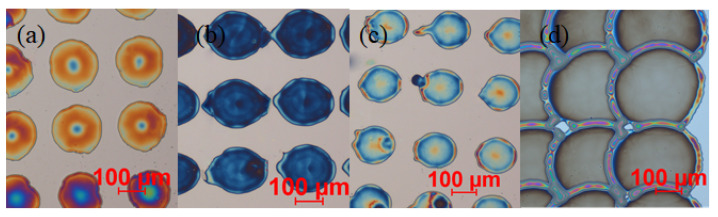
Micrographs of PEI dots deposited on differently prepared surfaces: (**a**) only standard cleaning, (**b**) dehydration bake, (**c**) HMDS pretreatment, (**d**) RIE O_2_ plasma treated.

As stated in the introduction, our primary goal was to establish the control over the thickness and deposition repeatability and precision of the functional films. Micrographs of the part of the PEI-functionalized CMUT surface are shown in Figure 4. The images (a–c) show the nearly circular pattern of the deposited droplets of the functional film, and the image in (d) shows a more continuous pattern established by joined droplets. In all cases, the same amount of 1% PEI/methanol solution was deposited (approximately 5 × 10^−15^ m${}^{3}$ ), with 250 μm pitch in both horizontal and vertical directions. The resulting difference of the functional film pattern was due to different surface preparation methods. In the case illustrated in (a), the CMUT surface underwent standard cleaning procedures in acetone, IPA, and DI water baths without any additional surface treatment, resulting with PEI dots with an average diameter of 160 μm. The cases illustrated in (b,c) show the PEI pattern of the samples, which additionally underwent dehydration bake (b) or HMDS treatment (c). In the case (b), the average diameter of the PEI dot was 197 μm, and in the case of (c), the average PEI dot diameter was measured at 150 μm. Surface treatment by oxygen plasma (80 W, 30 mTorr, 40 sccm O_2_, 60 s) after a standard cleaning gave the most consistent result illustrated in (d) with the overlapping PEI dots and average diameter of 360 μm.

One can conclude here that inkjet-printed PEI dots tend to form a mostly uniform and considerably thin functional coating in the case of increased hydrophilic surface properties, such as dehydration bake and oxygen plasma treatment. According to the light interference scale, the average thickness of the functional film was measured from 100 to 1000 nm (see the Experimental section for a detailed presentation of the thickness measurements), while the AFM scans were used to explore the surface morphology of the functional films at the nanoscale and verify the results of the optical thickness measurement.

**Figure 5:**
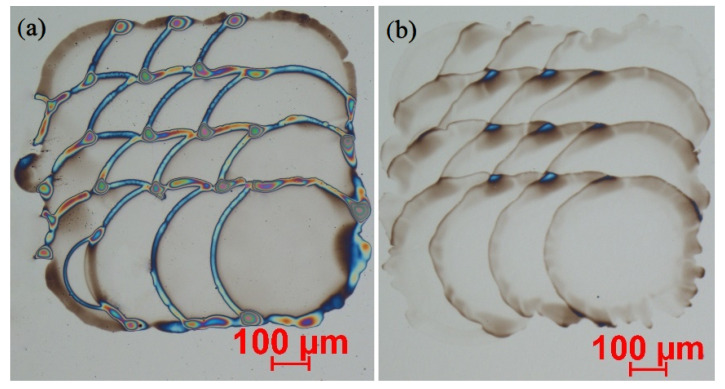
Comparison of inkjet-printed dots from differently thinned PEI solutions: (**a**) PEI dots printed from 1% *w*/*v* solution; (**b**) PEI dots printed from 0.3% *w*/*v* solution.

Next, we tested the effect of PEI dilution by comparing resulting functional films prepared from 1.0% and 0.3% *w*/*v* PEI/methanol solutions. In Figure 5, the patterns of PEI dots deposited on the oxygen plasma-treated surface for the corresponding dilution rate are shown. In the (a) part of the figure, one can see the dots printed from the 1.0% *w*/*v* solution, and the (b) part illustrates the dots resulting from the 0.3% *w*/*v* solution. In both cases, the printing was completed at the same step of 150 μm. Obviously, the solution of lower concentration (part b) allows better control over the thickness of the functional film, while the thicker solution (part a) tends to form considerably thick rings around the center part of the dots. According to the light interferometry and AFM measurements, the thickness of the deposited functional film may vary from 100 nm in the central part to more than a micrometer at the edges of the dot. Thickness measurements are explained in detail in the further experimental subsections.

**Figure 6:**
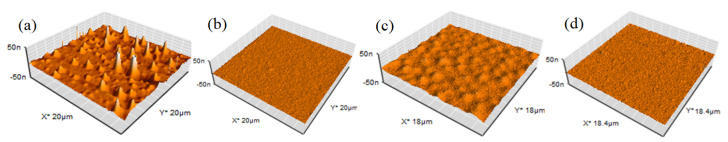
AFM scans of the deposited PEI dots over differenty prepared surfaces: (**a**) cleaned and untreated; (**b**) dehydration bake; (**c**) HDMS treated; (**d**) oxygen plasma treated.

Additionally, the morphology of the functional coating was explored by AFM at a 20 μm × 20 μm field of view at the dot center. Representative three-dimensional images of AFM scans of the samples illustrated in the optical micrographs (shown in Figure 4) are shown in Figure 6: (a) the surface of the PEI dot deposited over a cleaned but untreated surface; (b) dehydration bake; (c) HDMS treatment; and (d) oxygen plasma treatment.

We can summarize here that surfaces with expressed hydrophilic properties (b and d parts in Figure 4 and Figure 6) are favorable for a thinner and smoother PEI film surface, and the expressed hydrophobicity of the surface will lead to smaller but thicker dots of PEI with increased surface roughness. As thinner and smoother PEI dots enable layering of the functional film and give the possibility to produce the desirable film thickness by deposition of the corresponding number of single droplet layers, we have chosen the oxygen plasma surface treatment and 1.0% *w*/*v* to 0.3% *w*/*v* thinning of PEI solution as a standard protocol for our following experiments.

### 3.2. Functional Film Thickness Measurement through Light Interferometry and AFM Data

**Figure 7:**
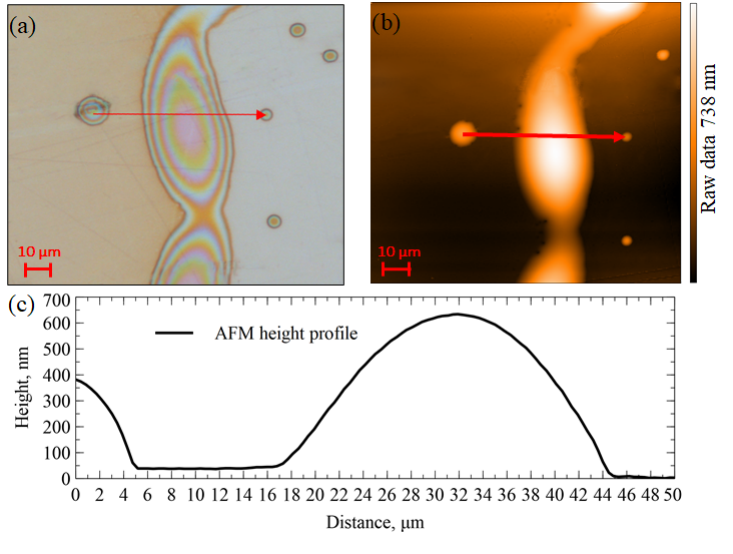
Illustration of AFM calibration of the thickness information carried by the light interference in optical microscopy images: (**a**) optical microscopy image of the PEI dot edge with a profile line marker; (**b**) AFM height map of the same area with the corresponding profile line marker; (**c**) AFM height profile along the line indicated in the (**a**,**b**) parts of the figure.

Due the transparency of the PEI films to the visible light, using the light interference pattern for the thickness measurements is possible. However, interference patterns need a reference height for the selected specific covering polymer and surface combination (PEI on gold) for which AFM was used. AFM alone is not a suitable tool for establishing polymer film thickness due to the small imaging area (110 μm by 110 μm for AFM vs. sensor area of 2 mm by 2 mm), limited scanning speed, polymer viscosity, and limited access to the absolute thickness values. Therefore, large field of view optical microscopy photographs were superimposed by the AFM data from 110 μm field of view to calibrate the film thickness information carried by the optical interference. A large proportion of CMUT PEI-functionalized device surfaces had even and level areas with lower order optical interference colors, and a relatively low proportion of the film area exhibited ring structures at the edges of PEI dots with a higher number of light interference lines (see Figure 5). Cropped optical microscopy images covering the active CMUT area were color-quantized to seven different colors by changing the image mode from RGB to indexed and selecting the custom seven-color palette using GIMP image editing software. For each functionalized device, the micrograph after each PEI layer deposition was taken, each color area, expressed in the number of pixels, was calculated, and this number was divided by the total number of pixels in the cropped image to obtain the fractional area occupied by each color. A large proportion of the film area exhibited up to three colors with a gradual proportional variation between themselves through the series of layer photographs. The edges of the PEI dots with a larger number of well-expressed interference lines were used for AFM calibration of the thickness information carried by the interference colors, as illustrated in Figure 7. The average PEI thickness of each CMUT active area was calculated as the area-normalized sum of the different thickness regions.

### 3.3. Functionalization and CO_2_ Sensitivity Tests

**Figure 8:**
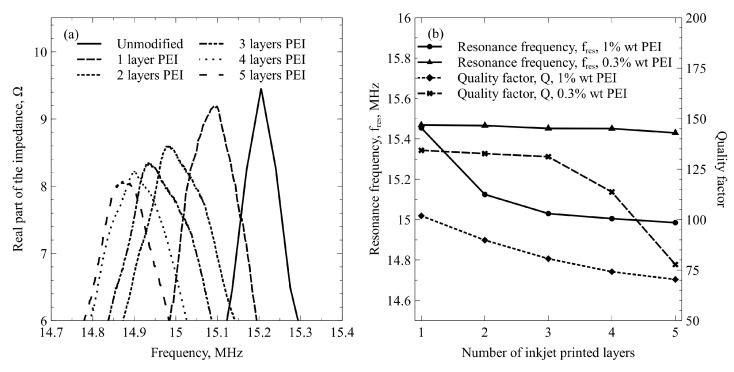
Functionalization shift in ambient air: (**a**) the frequency spectra of the real part of the CMUT electromechanical impedance before and after functionalization with an increasing number of inkjet-printed PEI layers of 1.0% *w*/*v* concentration; (**b**) resonance frequencies and quality factors of the sensors as a function of different numbers of inkjet-printed PEI layers.

The functionalization shift of a single device for various numbers of deposited layers of 1.0% *w*/*v* diluted PEI as measured in ambient air with approximately 40% relative humidity is illustrated in Figure 8. The resonance frequency peaks of the real part of CMUT electromechanical impedance shifted toward a lower frequency range. On devices with five inkjet-printed layers, they shifted by 0.4 MHz, which is a 2.5% change from the unmodified CMUT resonance value. At the same time, as can be seen from Figure 8b, for a five-layered PEI film printed from 1.0% *w*/*v* solution, the fractional resonance quality is lower by approximately 30%. As a contrast, functional films printed from 0.3% *w*/*v* PEI solution produce significantly lower functionalization shifts, especially at lower layer counts, which are attributable to the lower average thicknesses of the functional films. Thus, the resonance frequency shift produced by the functional films printed from 0.3% PEI solution is negligible even at five layers, and the shift of the resonance quality factor becomes significant only when more than three layers are deposited.

**Figure 9:**
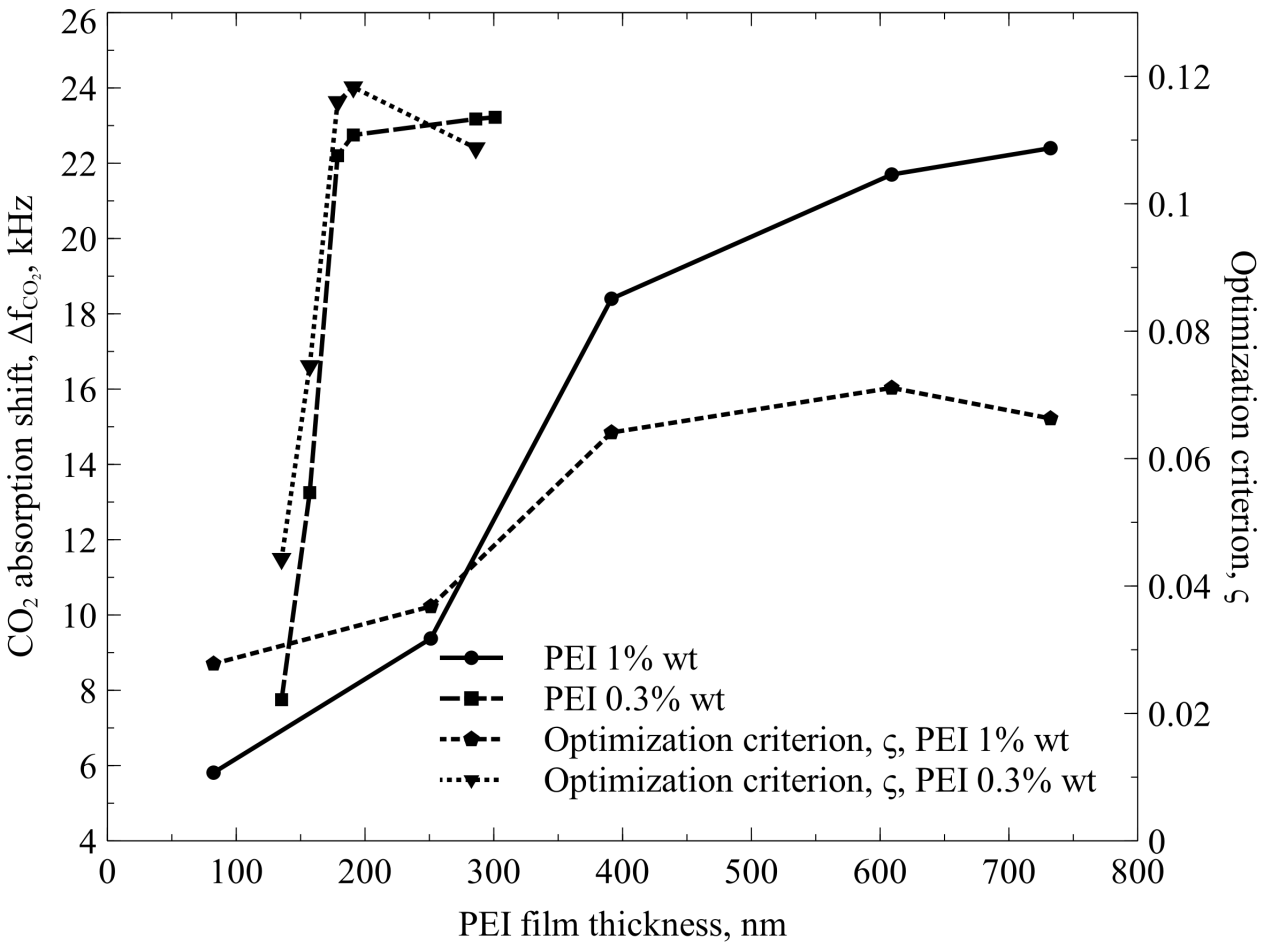
CO_2_ absorption shift and optimization criterion as functions of the average thickness of inkjet-printed PEI film. Different PEI solutions of 1.0% and 0.3% *w*/*v* in methanol, used for printing, are additional parameters.

To finalize the optimization research, we tested the sensor in a gas chamber by switching between pure N_2_ and CO_2_. In general, we followed our protocol already described in our previous research [12]. The measurement results are presented in Figure 9, where the absorption shift and optimization criterion for each version of the printed functional film are shown as functions of the average PEI film thickness. For each point, five to 10 measurements with different sensors were made. To estimate the measurement reliability, readings from the thickness measurements and from the absorption shift measurements were used to find the standard deviation at each data point. We found small deviations of the readings, which were attributable to the measurement noise and ranging not more than ±0.9% in both the vertical and horizontal axis. These deviations are too small to be shown on the graph.

The results illustrated in Figure 9 indicate that in the case of 1.0% *w*/*v* PEI solution, the maximum absorption shift $\Delta f_{SR}$ of approximately 22.3 kHz is reached when comparatively thick PEI films of 730 nm average thickness are printed, while the optimization criterion reaches its maximum at 600 nm thickness and is somewhat lower for the 730 nm case. At the same time, functional films printed from 0.3% *w*/*v* PEI solution demonstrate superiority, because they allow reaching the maximum absorption shift of 23.2 kHz at a 300 nm average thickness of the PEI film, and the optimization criterion reaches its maximum at less than 200 nm average thickness of the functional film. In this case, 200 nm can be assumed as the most effective average thickness of the functional film, where the entire volume of the film is used for CO_2_ absorption. Saturation of the absorption shift value for greater thicknesses is a good confirmation of this assumption.

In addition, this corresponds well to the results of our earlier research of the absorption mechanisms, indicating that several hundred nanometers is the credible limit of CO_2_ penetration depth [11]. The lower efficiency of the thicker functional films, prepared from 1.0% *w*/*v* PEI solution, can be explained by the lower sensitivity of the sensor, which is caused by the larger functionalization shift: CMUT cells become more damped due to the thicker edges of PEI dots, which overlap in the case of multi-layered functionalization.

**Figure 10:**
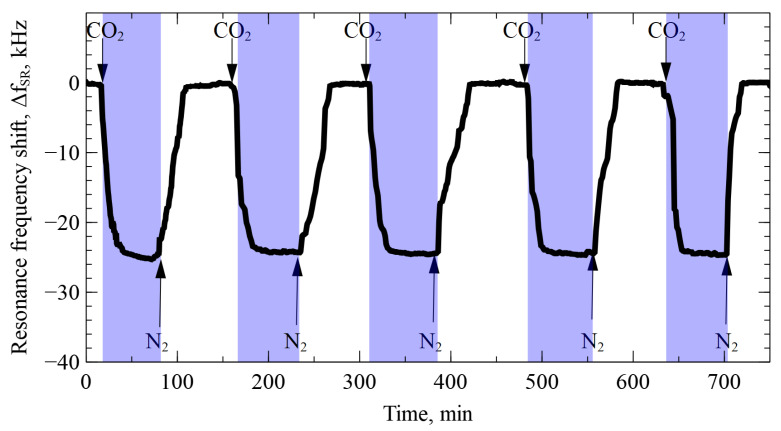
Transient plot of the sensor readings in response to the CO_2_ gas.

To demonstrate the stability of the sensor, we present the readings of $\Delta f_{SR}$ while repeatedly switching between pure N2 and pure CO2 for 12 h, as shown in Figure 10. From this graph, the stable and repeatable operation of the sensor can be observed.

## 4. Discussion and Conclusions

Inkjet-printed PEI films were proven to be fully functional for CO_2_ detection by the measurement of the PEI-functionalized CMUT electromechanical impedance. Switching from pure nitrogen to pure CO_2_ induced the frequency shift of more than 23.2 kHz (0.15% from nominal frequency), which was achieved with 190 nm thick functional film printed from 0.3% *w*/*v* PEI solution in methanol. Inkjet printing combined with an adequate surface preparation technique improved the control over CMUT functionalization by reducing the minimal functionalized area to 150 nm as the single PEI dot in the case of expressed hydrophobic CMUT surface properties and minimum average thickness of less than 100 nm in the case of hydrophilic surface properties. It was also revealed that changing the hydrophobic/hydrophilic surface properties prior to the deposition of PEI can result in a different nanometer-scale roughness of the functional film surface. It was also demonstrated that PEI diluted to 0.3% *w*/*v* is superior to the thicker solution of 1.0% *w*/*v* as a functional film precursor, because it provides smoother and more even distribution of the functional material and provides less damping of the CMUT cells. The functionalization shift of the sensors with 190 nm thick PEI film printed from 0.3% *w*/*v* solution was still negligible both for the resonance frequency and the resonance quality. A minor functionalization shift while the response to the gas switching was at the highest is the most important result of this research.
